# Somnal—A Hypnotic

**Published:** 1891-09

**Authors:** Irving D. Wiltrout

**Affiliations:** Physician in charge of the Holmes Sanitarium for Nervous Diseases, Hudson, Wis.


### Selections.

---

### SOMNAL—A HYPNOTIC.

By IRVING D. WILTROUT, M. D.,

Physician in charge of the Holmes Sanitarium for Nervous Diseases, Hudson, Wis.

Since I have been placed on the Committee on New Remedies, and in view of the fact that I am always desirous to keep up the interest of this Society by promptly responding to any task that may be set before me, I write this brief paper on a remedy that is not new to all of you, but some of you may not yet have tested its merits in that most annoying and often intractable symptom—insomnia.

The remedy I refer to is somnal. I show you here a sample of it. It is, as you see, a colorless liquid, resembling chloroform in its appearance and behavior when added to cold water, in which it forms globules, and refuses to mix or dissolve. When shaken with water the mixture is milky, but quickly separates. It is soluble in hot water and alcoholic solutions, and dissolves resinous substances and fats. The odor is rather delightful, and resembles somewhat that of spirits of nitrous ether. The taste is pungent, and for administration it needs free dilution. When whisky is not objectionable, or alcohol, it can be dissolved in either, to which water can then be added until the taste is not unpleasant. The taste can be disguised well in syrup of ginger or licorice.

Somnal is inflammable, and burns with a flame resembling alcohol. Somnal can be said to be a new remedy, for it was first brought to notice by Radlauer, of Berlin, in the Fall of 1889. It is formed by the union of chloral, alcohol, and urethrane; but it is not simply a mixture of these bodies. It differs from chloral-urethrane by the addition of $C_2H_4$, its formula being $C_7H_{12}Cl_3O_3N$. The dose ranges from fifteen to thirty drops. In its action it resembles chloral in quickness of effect and naturalness of the sleep produced. No marked depressing influence is exerted upon the pulse or respiration, though it is noticed that the breathing becomes slower and the pulse slower and fuller, as in natural sleep.

I have used this drug in upwards of thirty cases, and in no instance did I find any disagreeable after-effects. The head remains clear on waking, and the stomach unaffected. No constipating or relaxing effect follows its issue. The kidneys are slightly quickened. No increase of dose is called for, however long you use the remedy. Usually two doses are sufficient. I have the habit of giving the first dose at eight o'clock and the second at ten. A night's rest usually follows. In aggravated cases of insomnia I order a third dose administered at two A. M. if the patient is wakeful.

The sleep is very natural. It does not, like chloral, depress the heart, irritate the stomach and produce morning drowsiness, or disturb the gait, dull the sensibilities and irritate the stomach, which is often the case when sulfonal is used. In a form of insomnia which accompanies general neuralgic pains, this remedy almost invariably relieves the pain, and provokes a restful sleep.

In the fretfulness of nervous people who cannot sleep, as in cases of melancholia agitata, hysteria, hypochondria, and puerperal mania, I have found this remedy preferable to any other.

I have no experience in using this drug in the sleeplessness of children, nor have I witnessed its results in the acute febrile diseases. I believe that this remedy stimulates the gastric mucous membrane, and by so doing of tenrelieves nausea and pain, improves the appetite, and regulates the bowels.

Its power of relieving nausea and accumulation of gas in the stomach is very pronounced. I have in three instances administered it in small doses during the day for this purpose. The results were exceedingly satisfactory. As it is rapidly eliminated from the body, it may be administered each night or a number of days without any possibility of ill effects.

I am fond of old remedies; I take up new ones cautiously, but in my efforts to give refreshing and restful sleep to the sleepless and worn-out nervous cases that come under my care, I was ready to put this new remedy into immediate use, and I have done so with the results given above.—*N. W. Lancet.*

---

THERE is more than one leper to every 5,000 of the population in the vale of Kashmir. The entire number of inhabitants is only half a million.

---

AT THE WRONG BUSINESS.—Physician: "What is your profession, sir?" Patient (pompously): "I am a gentleman." Physician: "Well, you'll have to try something else; it does'nt agree with you."—*Life.*

---

ELECTROCUTION is the word coined to express legal death by electricity. Science, so-called, has invaded every domain, and now comes in to take the shame out of the legal death penalty. Is it science, though? and does not this innovation defeat the intent of criminal death punishment?—*N. C. Medical Journal.*

---

EDEMA OF THE GLOTTIS FOLLOWING INFLUENZA.—Before the Imperial Society of Medicine at Constantinople, Bavachi (*Gazette Med. d' Orient*, April 15, 1891,) reported three cases of influenza in which edema of the glottis developed. One was in a man of fifty, the second in a man of thirty, and the third in a girl of three. The first died, the general condition not being favorable for tracheotomy; the other two recovered without operative interference.

